# Escitalopram Dose Optimization During Pregnancy: A PBPK Modeling Approach

**DOI:** 10.3390/pharmaceutics17101341

**Published:** 2025-10-17

**Authors:** Seo-Yeon Choi, Eunsol Yang, Kwang-Hee Shin

**Affiliations:** 1 Research Institute of Pharmaceutical Sciences, College of Pharmacy, Kyungpook National University, Daegu 41566, Republic of Korea; chltjdus98@knu.ac.kr; 2 Department of Bioengineering and Therapeutic Sciences, University of California, San Francisco, CA 94143, USA; eunsol.yang@ucsf.edu; 3 Infectious Disease Healthcare, Kyungpook National University, Daegu 41566, Republic of Korea

**Keywords:** escitalopram, pregnancy, physiologically based pharmacokinetic, CYP2C19

## Abstract

**Background/Objectives**: Escitalopram, a first-line antidepressant, is primarily metabolized by CYP2C19. Its pharmacokinetics are altered during pregnancy. This study aims to predict maternal and fetal exposure to escitalopram during pregnancy and to propose safe and effective dosing strategies using physiologically based pharmacokinetic (PBPK) modeling. **Methods**: Predictive PBPK models for escitalopram were developed in nonpregnant women, pregnant women, and the fetoplacental unit using the Simcyp^®^ simulator. Additional models incorporating CYP2C19 phenotypes were constructed. Model performance was evaluated using visual predictive checks and by comparing predicted-to-observed ratios for the maximum plasma concentration (C_max_) and the area under the curve (AUC), within an acceptance criterion of 0.7–1.3. **Results**: Escitalopram concentrations at doses of 10–20 mg declined with advancing gestation. The cord-to-maternal concentration ratio was approximately 0.70 for both doses. Simulations of maternal and fetoplacental PBPK models across CYP2C19 phenotypes showed that most observed concentrations fell within the 95% confidence intervals of the predictions. Based on the therapeutic range attained and the maintenance of steady-state exposure, a once-daily 20 mg escitalopram dose was predicted to be appropriate during pregnancy. **Conclusions**: These findings suggest that a once-daily 20 mg dose appears optimal during pregnancy, highlighting the need to consider the gestational stage and CYP2C19 phenotype in dose optimization.

## 1. Introduction

Perinatal depression can arise from multiple factors, including the psychological and physiological changes in pregnancy and pregnancy-related complications [1]. Women with a history of depression potentially experience persistent symptoms during pregnancy [2]. Antenatal depression is among the most prevalent psychiatric complications during pregnancy, affecting approximately 20% of pregnant women [3]. However, the use of antidepressants during pregnancy is often limited owing to concerns regarding fetal safety. Nonetheless, untreated maternal depression poses a significant risk to both mother and fetus. Studies show that depression during pregnancy is related to increased risk of preterm birth and low birth weight, and in severe cases, may result in maternal suicide [4,5]. Therefore, effective treatment of perinatal depression is crucial to mitigate these adverse outcomes.

Pregnancy involves a series of physiological adaptations that support fetal development and prepare the maternal body for childbirth [6]. In singleton pregnancies, maternal weight typically increases by approximately 9–12 kg relative to pre-pregnancy levels [6]. Hemodynamic adaptations include elevated cardiac output and uterine blood flow, along with increases in plasma volume, total body fluid, and gestational tissues such as the placenta and amniotic fluid [6,7]. Furthermore, female sex hormone levels, particularly progesterone and estrogen, rise progressively throughout pregnancy [6].

Physiological adaptations during pregnancy influence drug pharmacokinetics. Altered gastric emptying, gastric pH, intestinal transit time, and bile acid secretion can modify oral bioavailability [8]. Additionally, increased total body water, body fat, and cardiac output, together with reduced plasma protein levels, may influence drug distribution and binding [9]. During pregnancy, CYP2D6 activity increases approximately threefold, CYP3A4 activity approximately twofold, while CYP2C19 activity decreases by approximately 50% [7,10]. Furthermore, increased renal blood flow, elevated glomerular filtration rate, and upregulation of renal transporters such as organic anion transporter 1 and 3 potentially enhance renal drug clearance [9].

Escitalopram, a selective serotonin reuptake inhibitor, is a first-line antidepressant for major depressive disorder, generalized anxiety disorder, and panic disorder [11]. The recommended therapeutic dose is 10 mg once daily, which can be increased to a maximum of 20 mg [12]. Its bioavailability is approximately 80%, with about 56% bound to plasma proteins [12]. Escitalopram undergoes extensive hepatic metabolism, with only approximately 7% excreted unchanged in urine [12]. The primary metabolic pathways involve CYP2C19 and CYP3A4, with CYP2D6 playing a lesser role [12]. Studies report phenotype-dependent variations in escitalopram pharmacokinetics. Average plasma concentrations are approximately 3.3-fold higher in CYP2C19 poor metabolizers (PMs) and 1.5-fold higher in intermediate metabolizers (IMs) than those in normal metabolizers (NMs), whereas ultra-rapid metabolizers (UMs) exhibited approximately 10% reduction [13]. Metabolites such as S-demethylcitalopram and S-didemethylcitalopram exhibit significantly lower pharmacological activity, approximately 7-fold and 27-fold lower than that of the parent compound, respectively, resulting in negligible serotonin reuptake inhibition [12]. 

Pharmacokinetic (PK) alterations of escitalopram during pregnancy have been reported, along with concerns regarding its safety, particularly in late pregnancy. One study shows that the plasma concentration-to-dose ratio of escitalopram at 6–8 weeks postpartum is approximately 63% higher than that at 36 weeks of gestation [14]. In addition to these PK findings, regulatory guidelines have highlighted potential safety concerns related to late-pregnancy administration of escitalopram. According to the U.S. Food and Drug Administration (FDA) prescribing information, escitalopram exposure during late pregnancy is related to an approximately twofold increase in the risk of postpartum hemorrhage [12]. Moreover, neonates may develop persistent pulmonary hypertension of the newborn and neonatal adaptation syndrome, warranting close monitoring [12]. Although the mechanism by which selective serotonin reuptake inhibitors influence pulmonary vasculature remains unclear, these safety concerns highlight the need to optimize escitalopram dosing during pregnancy [15].

Physiologically based pharmacokinetic (PBPK) modeling has emerged as a quantitative approach for predicting PK alterations and evaluating potential safety risks [16]. This method is particularly valuable in populations where clinical trials pose ethical challenges, such as pregnant women and fetuses [17]. Although PBPK modeling has been applied to predict escitalopram pharmacokinetics in elderly patients, its application in pregnancy remains limited [18].

Selective serotonin reuptake inhibitors (SSRIs) are commonly prescribed antidepressants during pregnancy, with sertraline and fluoxetine being the most frequently reported agents [19]. Escitalopram, a member of the SSRI class, has not been reported to be prescribed as frequently as sertraline and fluoxetine in pregnant women [19]. However, a clinical study demonstrates that escitalopram crosses the placenta substantially, with median cord-to-maternal ratios around 0.73 (range 0.71–0.91) [20]. This indicates considerable fetal exposure and highlights the need for quantitative approaches to characterize its disposition during pregnancy. Despite its clinical relevance, evidence on fetal transfer and pregnancy-related pharmacokinetic changes remains limited [21]. PBPK modeling of escitalopram during pregnancy has not been established. Therefore, the study aims to develop PBPK models for predicting maternal and fetal escitalopram concentrations throughout pregnancy and to propose a safe and effective dosing strategy.

## 2. Materials and Methods

### 2.1. Software and Workflow

**Figure 1:**
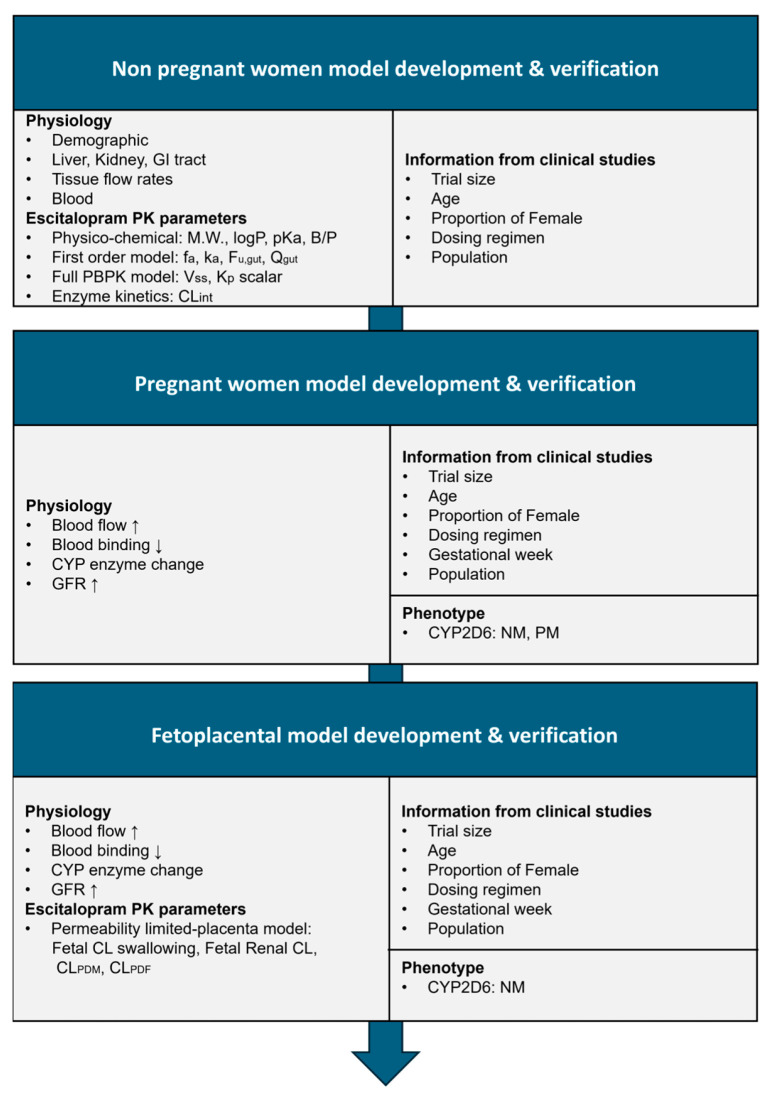
Workflow for developing the escitalopram PBPK model. Upward arrows denote an increase, and downward arrows denote a decrease. Abbreviations: M.W., molecular weight; LogP, logarithm of octanol/water partition coefficient; pK_a_, negative logarithm of acid dissociation constant; B/P, blood-to-plasma ratio; fa, fraction absorbed; k_a_, absorption rate constant; f_u,gut_, fraction unbound in gut enterocytes; Q_gut_, Hybrid parameter representing drug absorption rate from the gut lumen, removal of drug from the enterocyte by enterocytic blood supply, and enterocyte volume; Vss, steady-state volume of distribution; K_p_ scalar, tissue-to-plasma partition coefficient scalar; CL_int_, intrinsic clearance; CYP, cytochrome P450; GFR, glomerular filtration rate; NM, normal metabolizer; PM, poor metabolizer; CL_PDM_, Maternal-placental barrier clearance; CL_PDF_, placental-fetal barrier clearance.

The PBPK model of escitalopram was developed using Simcyp^®^ PBPK simulator version 21 (Certara, Princeton, NJ, USA) to simulate pharmacokinetics in nonpregnant women, pregnant women, and the fetoplacental unit. Figure 1 shows the overall workflow. Initially, a predictive model for nonpregnant women was developed by integrating drug-specific physicochemical properties with absorption, distribution, and elimination parameters, along with clinical study designs derived from observed studies [20,22,23,24,25,26]. This model was subsequently validated using data extracted from published literature. Using the validated nonpregnant model, pregnancy-specific models were generated by incorporating physiological adaptations of pregnancy and subsequently validated. A fetoplacental unit model was then integrated, including fetal-related PK parameters. Finally, predictive models incorporating CYP2C19 phenotypes were developed for both pregnant women and the fetoplacental unit. Predictive performance across all models was evaluated based on the inclusion of observed data within the predicted 95% confidence intervals, prediction ratios, and the absolute average fold error (AAFE).

### 2.2. Development of the Nonpregnant Women Model

Table 1 presents the PK parameters of escitalopram applied in the simulation. The blood-to-plasma ratio (B/P), fraction absorbed (f_a_), fraction unbound in gut enterocytes (fu,gut), enzyme kinetics parameters, and renal clearance (CL_R_) values were obtained from published literature [27,28,29]. The absorption rate constant (k_a_), hybrid parameter representing drug absorption rate from the gut lumen, removal of drug from the enterocyte by enterocytic blood supply, and enterocyte volume (Q_gut_), and tissue-to-plasma partition coefficient scalar (K_p_ Scalar) were optimized using the parameter estimation module in the Simcyp^®^ simulator, based on observed plasma concentration-time data in healthy participants [23]. Parameter estimation was conducted using a weighted least squares objective function with the Nelder–Mead minimization method, and the optimization procedure was repeated 100 times to obtain the parameter values. The K_p_ Scalar was applied to predict the steady-state volume of distribution (V_ss_) using the Rodger and Rowland method (Table 1).

Clinical data were used to develop and evaluate the nonpregnant women model. The trial size was determined based on the number of participants reported in the literature, and simulations were repeated 10 times [23,26]. Although the original studies involved healthy individuals aged 18–45 years, the population was modified to represent nonpregnant women designated at gestational week 0, while maintaining the same age range to align with the source data [23,26]. Escitalopram dosing regimens were derived from the literature and included a single 20 mg dose or repeated daily doses of 10 mg or 30 mg [23,26]. Although the referenced studies administered 10 mg for 24 days and 30 mg for 18 days, steady-state concentrations are reported to be achieved within approximately 7 days according to the FDA prescribing information for escitalopram [12]. Therefore, a 10-day repeated dosing regimen was applied in the simulations.

### 2.3. Development of the Pregnant Women Model

Physiological parameters were incorporated into the PBPK model using a gestational age-dependent equation provided by the Simcyp^®^ simulator. The parameters included plasma protein binding, hepatic CYP enzyme activity, renal function, and blood flow. Each parameter was scaled continuously with gestational age (see Equation (1)) [17]:

GW_0_ ∙ (1 + B_1_∙ GW + B_2_∙ GW^2^ + B_3_∙ GW^3^ + B_4_∙ GW^4^) (1)

GW_0_ denotes the baseline value at gestational week 0, while B_1_, B_2_, B_3_, and B_4_ are coefficients representing gestational age–dependent changes in physiological parameters, with each corresponding to a successive power of gestational week (GW). GW serves as the independent variable for modeling physiological adaptations during pregnancy. CYP2C19 activity was assumed to remain constant across gestation, with all coefficients (B_1_–B_4_) set to zero. In contrast, CYP2D6 and CYP3A4 activities were scaled with gestational age based on the default Simcyp^®^ settings, with coefficients of B_1_ = 0.0163 and B_2_ = 0.0009 for CYP2D6, and B_1_ = 0.0129 and B_2_ = 0.0005 for CYP3A4. 

Clinical data were used to evaluate the pregnant women model [20,24,25]. Owing to the limited sample size, data were pooled for simulation when identical doses were administered within the same trimester. However, data across different trimesters were not pooled, even at similar doses, to account for potential gestational age–related differences in pharmacokinetics. Consequently, a pooled simulation was performed only for the third trimester, with gestational age set at 35 weeks, representing the median of the third trimester (29–40 weeks). The virtual population age range was set at 18–45 years or 32–43 years to encompass the ranges reported in the referenced studies [20,24,25]. The trial size generated by the Simcyp^®^ simulator was applied. Dosing regimens included 10 mg and 20 mg of escitalopram administered once daily [20,24,25]. All repeated-dose regimens were simulated for 10 days, consistent with studies in which escitalopram concentrations were assessed at steady state.

### 2.4. Development of the Fetoplacental Model

A fetoplacental model was incorporated into the pregnancy model integrating fetal-specific PK parameters. In the Simcyp^®^ simulator, the fetoplacental unit is represented using a permeability-limited placental model implemented from gestational week 15. This framework includes two sequential barriers: the maternal-placental barrier (CL_PDM_) and placental–fetal barrier (CL_PDF_), with permeability parameters estimated based on villous surface area and physicochemical properties including hydrogen bond donor count and polar surface area [30]. This model simulates placental drug transfer into the fetal circulation, thereby facilitating the evaluation of fetal drug exposure during pregnancy. Fetal clearance through swallowing (L/h/kg fetal weight) was calculated using Equation (2) [31], based on the average volume of amniotic fluid ingested by a full-term fetus (750 mL) and the average fetal weight (3.7 kg) (see Equation (2)) [32,33]. Fetal CL_R_ (L/h/kg fetal weight) was calculated using Equation (3) [31], which incorporated adult CL_R_ (4.0 L/h), fetal glomerular filtration rate (GFR, 4.92 mL/min), adult GFR (121 mL/min), and fetal weight (see Equation (3)) [33,34,35]. Table 1 presents the details of these clearance values.

(2) $$\mathrm{Fetal}\;\mathrm{CL}\;\mathrm{swallowing}=\frac{\mathrm{Fetal}\;\mathrm{Swallowing}\;\mathrm{amniotic}\;\mathrm{volume}\;(\frac{\mathrm{mL}}{\mathrm{day}})}{24\;h/\mathrm{day}}\;\times \;\frac{1\;L}{1000\;\mathrm{mL}}\;\times \;\frac{1}{\mathrm{Fetal}\;\mathrm{weight}\;(\mathrm{kg})}$$

(3) $$\mathrm{Fetal}\;\mathrm{renal}\;\mathrm{CL}=\frac{\mathrm{Adult}\;{\mathrm{CL}}_{R}\;(L/h)\;\times \;\mathrm{Fetal}\;\mathrm{GFR}\;(\frac{\mathrm{mL}}{min})}{\mathrm{Fetal}\;\mathrm{body}\;\mathrm{weight}\;(\mathrm{kg})\;\times \;\mathrm{Adult}\;\mathrm{GFR}\;(\frac{\mathrm{mL}}{min})}$$

Maternal-placental and placental-fetal barrier clearances were predicted using the Simcyp® simulator, based on polar surface area and hydrogen bond donor properties (Table 1) [28].

The fetoplacental predictive model [20,24] was evaluated against clinical data from pregnant women aged 18–45 years. Trial size was set to the Simcyp^®^ default of 10 × 10, owing to the limited sample size. Dosing regimens included 10 mg and 20 mg of escitalopram once daily for 10 days [20,24]. Gestational age ranged from 39–40 weeks [20,24].

### 2.5. Development of Pregnant Women and Fetoplacental Model Based on CYP2C19 Phenotypes

Clinical data were used to evaluate the pregnant women and fetoplacental predictive model stratified by CYP2C19 phenotype [22]. CYP2C19 genetic variability was incorporated by adjusting phenotype frequencies in the Simcyp^®^ pregnancy population, whereas fetal phenotypes were excluded because the platform does not support their implementation. Owing to the limited sample size, data were pooled for simulation when identical doses were administered within the same trimester. The virtual population was defined with an age range of 31–39 years, and the trial size was set to the Simcyp^®^ default of 10 × 10 [22]. Dosing regimens included escitalopram at 10 mg and 20 mg once daily [22]. All repeated dosing regimens in the simulation were standardized to 10 days, based on studies in which escitalopram concentrations were assessed at steady state. Gestational age was defined according to the data pooling criteria. For the third trimester, 35 weeks was applied as the median of the reported range (29–40 weeks). In contrast, for CYP2C19 NM data, all observations were pooled at a uniform gestational age, and 40 weeks was therefore used [22].

To optimize escitalopram dosing across CYP2C19 phenotype-defined populations, the predicted concentration–time profiles were integrated into multiple population simulations, with separate simulations presented for each gestational trimester. The trial size was set to 20 × 20, corresponding to the 10 × 10 trial size applied to each individual model.

### 2.6. Evaluation of the Physiologically Based Pharmacokinetic Model

The predictive performance of the PBPK model was evaluated by determining whether the observed mean plasma concentration-time profiles fell within the predicted 95% confidence intervals. The prediction ratios for key pharmacokinetic parameters, including maximum plasma concentration (C_max_) and area under the curve (AUC), were calculated using Equation (4):

(4) $$\mathrm{Prediction}\;\mathrm{ratio}\;=\;\frac{\mathrm{Predicted}}{\mathrm{Observed}}$$

Model performance was considered acceptable when prediction ratios were within the 0.7–1.3 range [36]. In addition, AAFE for Cmax and AUC was calculated using Equation (5):

(5) $$\mathrm{AAFE}\;=\;10^{\frac{\sum \left|\log(\frac{\mathrm{Predicted}}{\mathrm{Observed}})\right|}{n}}$$

where n denotes the total number of observed-predicted data pairs corresponding to the number of mean concentrations, or in the case of exposure parameters (Cmax, AUC), the number of profiles included in the calculation. An AAFE < 2 was considered indicative of acceptable model performance [37].

### 2.7. Sensitivity Analysis of the Physiologically Based Pharmacokinetic Model

Global sensitivity analysis using the Morris method was conducted to quantify the influence of nine drug-specific parameters in the nonpregnant models on predictions of AUC and Cmax. High absolute mean (µ*) values enable the ranking of parameters, with larger µ* values signifying greater influence on model outputs [38].

### 2.8. Dose Optimization Strategy

The therapeutic range of escitalopram is reported as 15–80 ng/mL, based on steady-state trough concentrations [39]. To evaluate optimal therapeutic dosing across pregnancy trimesters and CYP2C19 phenotypes, the predicted plasma concentration–time profiles were compared with this therapeutic range. Additionally, steady-state average concentration (C_avg,ss_) and steady-state maximum concentration (C_max,ss_) were summarized to provide complementary insights into exposure, with a threshold of ≥17 ng/mL applied for interpreting C_avg,ss_ [40,41,42].

## 3. Results

### 3.1. Development and Verification of the Escitalopram Prediction Model in Nonpregnant Women

Figure S1 illustrates the plasma concentration–time profiles of escitalopram in nonpregnant women. The observed data fell within the 95% prediction intervals of the predicted plasma concentration-time curves (Figure S1). Furthermore, the predicted ratios for C_max_ and AUC ranged from 0.79 to 0.83 and from 0.91 to 1.14, respectively, all of which fell within the acceptable range of 0.7 to 1.3 (Table S1). Additionally, all AAFE values were <2, thereby indicating acceptable model performance (Table S1). Sensitivity analyses revealed that C_max_ predominantly influenced through the K_p_ scalar, f_a_, and k_a_, while AUC was mainly affected through f_a_, CYP2D6 intrinsic clearance (CL_int,2_), and CYP2C19 intrinsic clearance (CL_int,3_) (Figure S2).

### 3.2. Development and Verification of the Escitalopram Prediction Model in Pregnant Women

**Figure 2:**
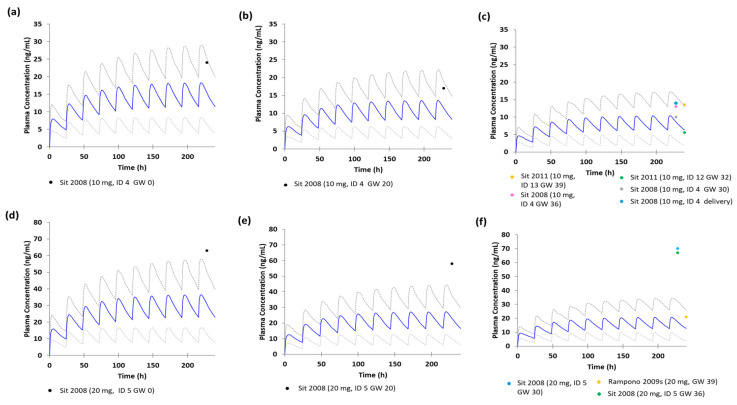
Predicted plasma concentration–time profiles of escitalopram in pregnant women following (**a**–**c**) multiple oral doses of 10 mg, and (**d**–**f**) 20 mg at (**a**,**d**) gestational week 0, (**b**,**e**) gestational week 20, and (**c**,**f**) gestational week 35. Blue line represents the mean predicted plasma concentration, and gray dotted lines indicate the 5th and 95th percentiles of predicted plasma concentrations. Circles indicate observed plasma concentrations. Abbreviation: GW, gestational week.

Figure 2 shows the plasma concentration-time profiles of escitalopram after multiple 10 or 20 mg oral doses at gestational weeks 0, 20, and 35. A proportion of observed concentrations fell within the 95% prediction intervals (pooled AAFE = 1.90), indicating acceptable accuracy. However, approximately 40% of ID 5 data points were outside the intervals. These findings indicate that although the overall predictive performance of the model was acceptable, it did not fully capture interindividual variability.

Table 2 presents the predicted pharmacokinetic parameters of escitalopram in pregnant women. Across both dose levels, advancing gestational age from week 0 to week 35 resulted in a 43.1% decrease in C_max_ and a 43.9% reduction in AUC.

### 3.3. Development and Verification of the Escitalopram Prediction Model in the Fetoplacental Unit

Figure S3 illustrates the predicted concentration-time profiles of escitalopram in umbilical cord blood at term, following multiple oral doses of 10 mg and 20 mg. All observed values were within the 95% confidence intervals of the predicted profiles and the pooled AAFE was 1.66, indicating that the model performance is acceptable.

Table S2 summarizes the predicted pharmacokinetic parameters of escitalopram in umbilical cord blood following oral administration of 10 mg and 20 mg doses. The predicted cord-to-maternal plasma concentration ratios were 0.71 and 0.69 for the 10 mg and 20 mg doses, respectively.

### 3.4. Development and Verification of the Escitalopram Prediction Model in Pregnant Women and the Fetoplacental Unit Based on CYP2C19 Phenotypes

**Figure 3:**
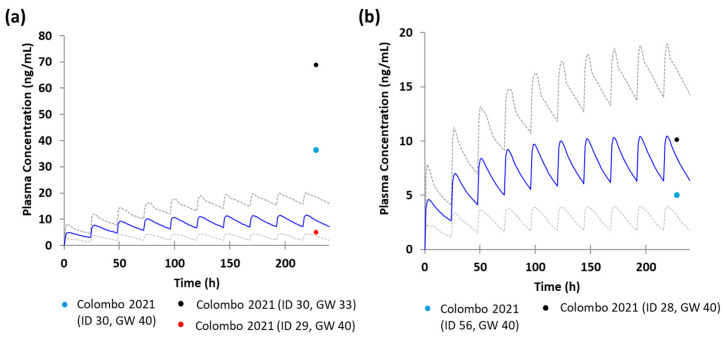
Predicted plasma concentration–time profiles of escitalopram in pregnant women following multiple oral doses of 10 mg, stratified using CYP2C19 phenotype: (**a**) IM at gestational week 35 and (**b**) NM at gestational week 40. Blue line indicates the mean predicted plasma concentration, while gray dotted lines indicate the 5th and 95th percentiles. Circles indicate observed plasma concentrations. Abbreviations: IM, intermediate metabolizer; NM, normal metabolizer; GW, gestational week.

Figure 3 illustrates the predicted plasma concentration-time profiles of escitalopram following multiple oral doses of 10 mg at gestational week 35 for CYP2C19 IM and at week 40 for CYP2C19 NM. A proportion of observed concentrations fell within the 95% prediction intervals; however, some data points (ID 30) were outside the prediction interval (Figure 3). The pooled AAFE was 2.07, exceeding the acceptable criterion (AAFE < 2). Table 3 summarizes the predicted pharmacokinetic parameters at gestational week 35 for IM and week 40 for NM.

According to clinical study data, Figure S4 illustrates the predicted concentration–time profile of escitalopram in umbilical cord blood at term for pregnant women with a CYP2C19 NM phenotype, following multiple oral doses of 10 mg. The observed concentration fell within the 95% confidence interval of the predicted profile. In addition, the pooled AAFE was 1.69, demonstrating that the model exhibits acceptable predictive performance.

Table S3 summarizes the predicted pharmacokinetic parameters of escitalopram in umbilical cord blood following oral doses of 10 mg in pregnant women with a CYP2C19 NM phenotype. The predicted cord-to-maternal plasma concentration ratio was 0.70.

### 3.5. Dose Optimization of Escitalopram in Pregnant Women Based on Model Predictions

**Figure 4:**
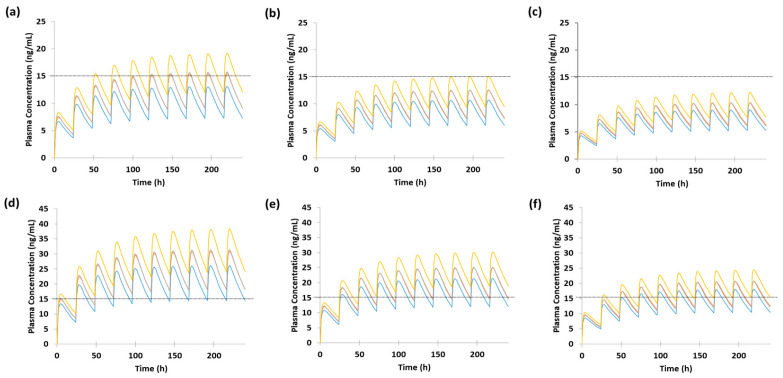
Dose optimization of escitalopram during pregnancy based on CYP2C19 phenotypes. Predicted plasma concentration–time profiles following (**a**–**c**) 10 mg, and (**d**–**f**) 20 mg oral doses at (**a**,**d**) gestational week 7, (**b**,**e**) gestational week 21, and (**c**,**f**) gestational week 35. Light blue line: UM; orange line: NM; gray line: IM; yellow line: PM. The therapeutic range of escitalopram is 15–80 ng/mL. Abbreviations: UM: ultrarapid metabolizer; NM: normal metabolizer; IM: intermediate metabolizer; PM: poor metabolizer.

Following a 10 mg dose, the predicted C_min,ss_ ranged from 5.23 ng/mL to 12.0 ng/mL, remaining below the therapeutic range across all CYP2C19 phenotypes. Similarly, the predicted C_avg,ss_ (7.2–15.9 ng/mL) did not reach the 17 ng/mL threshold associated with serotonin transporter (SERT) occupancy (Figure 4, Table S4).

Following a 20 mg dose, the predicted C_min,ss_ ranged from 10.5 ng/mL to 24.1 ng/mL, reaching or approaching the therapeutic range in most cases, except during the second and third trimesters of CYP2C19 UM and the third trimester in NM and IM. Additionally, the predicted C_avg,ss_ was within or close to the 17 ng/mL threshold across all phenotypes, except the third trimester in CYP2C19 UM (Figure 4, Table S4).

## 4. Discussion

In this study, we evaluated the influence of pregnancy-induced physiological changes and CYP2C19 phenotype variability on escitalopram exposure in both mothers and fetuses. PBPK modeling was used to predict maternal plasma and umbilical cord concentrations of escitalopram during pregnancy. Additionally, a pregnancy–fetoplacental model incorporating CYP2C19 phenotypes was developed. Based on these models, optimal escitalopram dosing regimens during pregnancy were proposed, with specific consideration of CYP2C19 phenotypic differences.

Pregnancy-induced changes in CYP enzyme activity potentially contributed to the observed reduction in escitalopram exposure. In the maternal PBPK model, increasing gestational age from week 0 to week 35 resulted in a 43.1% decrease in C_max_ and a 43.9% decrease in AUC for escitalopram (Figure 2). Studies report that during pregnancy, CYP2D6 activity increases approximately threefold, CYP3A4 activity approximately twofold, while CYP2C19 activity decreases by approximately 50% [7,10]. Despite reduced CYP2C19 activity, a clinical study reports a 23% higher desmethylcitalopram-to-citalopram metabolic ratio during pregnancy than during postpartum, indicating increased metabolism [43]. Although N-demethylation of citalopram involves CYP2C19, CYP2D6, and CYP3A4, the didesmethylcitalopram-to-desmethylcitalopram ratio, a specific marker of CYP2D6 activity, was 54% higher during pregnancy, suggesting CYP2D6 induction [43]. In contrast, no evidence suggests a comparable pregnancy-induced increase in CYP3A4-activity [43]. These findings indicate that CYP2D6 induction, despite reduced CYP2C19 activity, predominates in the increased citalopram N-demethylation and may contribute to reduced escitalopram exposure during pregnancy [43]. Consistent with these findings, sensitivity analyses conducted in the nonpregnant model showed that systemic exposure was most influenced by fa, CYP2D6 intrinsic clearance, and CYP2C19 intrinsic clearance (Figure S2). Collectively, these findings suggest that pregnancy-associated modifications in CYP enzyme activity represent a major factor contributing to the reduced escitalopram exposure during pregnancy.

Genetic polymorphisms in CYP enzymes represent a major determinant of interindividual variability in escitalopram plasma concentrations [44,45]. The pregnancy model, adapted from the nonpregnant women model, accurately predicted most observed concentrations within the 95% confidence interval. However, for subject ID 5, following a 20 mg dose, the predicted concentration was outside this interval (Figure 2). CYP enzyme genotypes are closely associated with individual metabolic capacity and variability in drug concentrations. Average escitalopram concentrations are approximately 3.3-fold higher in CYP2C19 PM and 1.5-fold higher in IM than in NM, while UM exhibits an approximate 10% decrease [13]. 

In this study, the genotypic distribution of the Caucasian pregnant population provided by the Simcyp^®^ simulator was incorporated, corresponding to the ethnicity of subject ID 5. The reported frequencies of PM for CYP2C19 and CYP2D6 are approximately 2% and 7%, respectively, while the frequency of CYP3A4 PM is approximately 0%, with IM reported at approximately 11% [46]. Based on these frequencies, CYP2C19 and CYP2D6 were classified as PMs and CYP3A4 as an IM. This configuration produced predicted concentrations within the 95% confidence intervals, with a pooled AAFE of 1.25 (Figure S5), suggesting that PBPK modeling with genotype information enhances the predictive accuracy of escitalopram plasma concentrations. However, since genotype data for ID 5 were unavailable, interpreting this participant as a PM remains speculative. Therefore, these findings should be considered exploratory rather than confirmatory.

The fetus is exposed to approximately 70% of the maternal escitalopram concentrations. The predicted placental clearance (CL_PDM_ and CL_PDF_ = 0.809) showed acceptable agreement with ex vivo cotyledon perfusion data for escitalopram (clearance index at 180 min, CI_180_ = 1.03), with an AAFE of 1.27, supporting model robustness [21]. At delivery, the fetoplacental model estimated cord-to-maternal plasma concentration ratios of 0.71 and 0.69 after 10- and 20-mg doses, respectively (Table S2), consistent with a previously reported value of 0.73 [20]. Another study reports a cord-to-maternal ratio of 0.49 for escitalopram [22], which also reasonably agrees with the fetoplacental model (pooled AAFE = 1.43). The moderate lipophilicity (logP = 1.34) and low molecular weight (324.39 g/mol) of escitalopram facilitate placental transfer, underscoring the need to consider fetal exposure when prescribing escitalopram during pregnancy [47,48]. While clinical data, mostly obtained at delivery, cannot confirm whether this ratio changes across gestation, fetal safety remains a concern. Since the major metabolites have negligible pharmacological activity compared with escitalopram, their clinical impact is likely minimal [12]. Therefore, optimizing the maternal dose to achieve the lowest effective exposure may help reduce unnecessary fetal exposure while maintaining therapeutic efficacy.

PBPK models incorporating CYP2C19 phenotypes (NM and IM) showed strong predictive performance, with most observed concentrations falling within the 95% confidence interval. However, subject ID 30 exhibited plasma concentrations above the predicted range (Figure 3). Incorporating CYP2D6 and CYP3A4 phenotypes into additional models did not resolve this discrepancy, suggesting that other physiological factors may contribute to interindividual variability. The AUC of escitalopram increases by approximately 51% and 69% in patients with mild and moderate hepatic impairment, respectively, while renal impairment reduces systemic clearance by approximately 17% [12]. The clinical data used in this study were not controlled for physiological variables, such as underlying comorbidities, which potentially contribute to the observed interindividual variability.

The therapeutic range of escitalopram is defined by a trough plasma concentration of 15–80 ng/mL, while the laboratory alert level is 160 ng/mL, indicating a threshold for potential clinical concern [39]. In this study, C_min,ss_ was evaluated using the lower limit of the therapeutic range (15 ng/mL) as a reference. Additionally, C_avg,ss_ and C_max,ss_ were assessed as supportive exposure metrics. Positron emission tomography studies indicate that SERT occupancy exceeds 80% at plasma concentrations ≥17 ng/mL, and that SERT binding in key brain regions (e.g., habenula, subgenual cingulate cortex, amygdala–hippocampus complex) may predict therapeutic response [40,41,42]. These findings suggest that maintaining drug exposure above a certain threshold is critical for pharmacological efficacy, highlighting the relevance of C_avg,ss_ in maintaining antidepressant effects.

The optimal escitalopram dose during pregnancy was predicted as 20 mg/day. For a 10 mg dose, the predicted C_min,ss_ values ranged from 5.23 ng/mL to 12.0 ng/mL across different CYP2C19 phenotypes, indicating subtherapeutic exposure in all cases. Similarly, C_avg,ss_ did not reach the 17 ng/mL threshold (Figure 4; Table S4). These findings suggest that a 10 mg/day dose may be insufficient during pregnancy. In contrast, at a 20 mg dose, the predicted C_min,ss_ values reached or slightly fell below the therapeutic range, except during the second and third trimesters for CYP2C19 UM and the third trimester for NM and IM. C_avg,ss_ values reached or approached the 17 ng/mL threshold across all phenotypes and trimesters, except for CYP2C19 UM in the third trimester. Collectively, these results highlight the potential need for adjusting escitalopram dosing during pregnancy.

To further explore escitalopram exposure based on genetic polymorphisms, CYP2C19 and CYP2D6 phenotype combinations at gestational week 35 were analyzed as clinically relevant configurations. Although escitalopram is primarily metabolized by CYP2C19 and CYP3A4, CYP2D6 activity nearly threefold during pregnancy and shows substantial genetic variability, with both PM and UM present in the population [7,10,12,46]. The results showed that CYP2C19 UM combined with CYP2D6 UM fell below the therapeutic range, whereas CYP2C19 PM combined with CYP2D6 PM remained within this range (Table S5 and Figure S6). These findings indicate that drug exposure may vary based on genotype even within the same trimester, highlighting the need for regular clinical monitoring.

This study has two primary limitations. First, although the PBPK model incorporated various CYP2C19 phenotypes, the simulations were based on limited phenotype data (EM/NM) without comprehensive individual genotype data, and fetal CYP2C19 phenotypes were not considered. Moreover, gestational changes in maternal CYP2C19 activity during pregnancy were not incorporated, potentially limiting the applicability of the model across diverse clinical populations. Second, the proposed dosing strategies were derived solely from simulation outcomes informed by limited observed data; therefore, these findings may be considered preliminary, necessitating further clinical validation before implementation in clinical practice. Despite these limitations, this study provides valuable insights into maternal and fetal escitalopram exposure, highlighting the potential utility of PBPK modeling in supporting optimal dosing strategies during pregnancy.

## 5. Conclusions

In this study, the PBPK model predicts that a once-daily 20 mg dose of escitalopram maintains therapeutic exposure during pregnancy. However, this interpretation should be approached with caution, particularly in the context of CYP enzyme polymorphisms and during the third trimester, when CYP3A4 and CYP2D6 activities reach their peak. These recommendations should be considered preliminary and require further clinical validation across diverse populations prior to clinical implementation. The model does not account for specific sources of variability, including individual genotypes, fetal phenotypes, and gestational changes in CYP2C19 activity. However, these findings indicate the importance of adjusting the doses to account for gestational physiological changes and genetic polymorphisms in drug-metabolizing enzymes. PBPK modeling serves as a valuable tool for supporting optimal dosing strategies and promoting the safe and effective administration of escitalopram in pregnant women. 

## Figures and Tables

**Table 1:** Input parameters for the escitalopram PBPK model.

Parameters	Values	References
Mol. weight (g/mol)	324.39	-
LogP_o:w_	1.34	Drugbank online Escitalopram
Compound type	Monoprotic Base	-
pK_a_	9.5	Drugbank online
B/P	2.0	[27]
fu_p_	0.44	[29]
Absorption model	First-order model	-
f_a_	1.0	[29]
ka, (1/h)	0.19	Parameter estimation
fu_gut_	1.0	[28]
Q_gut_	5.69	Parameter estimation
Distribution model	Full PBPK model	-
V_SS_ (L/kg)	13.513	Predicted by the Simcyp^®^ simulator
Kp Scalar	0.92	Parameter estimation
Enzyme kinetics parameters	CLint (μL/min/pmol of isoform): CYP2C19: 0.774 CYP2D6: 0.505 CYP3A4: 0.0155	[29]
CL_R_(L/h)	4.0	[29]
Transport (Permeability Ltd. organ)	Permeability limited- placenta model	-
Fetal CL swallowing (L/h/kg fetal weight)	0.00844	Calculated by Equation (2) (see methods in Section 2.4)
Fetal CL_R_ (L/h/kg fetal weight)	0.044	Calculated by Equation (3) (see methods in Section 2.4)
CL_PDM_ and CL_PDF_	0.80902	Predicted by the Simcyp^®^ simulator

Abbreviations: Mol., molecular; LogP_o:w_, logarithm of octanol/water partition coefficient; pK_a_, negative logarithm of acid dissociation constant; B/P, blood to plasma ratio; fu_p_: fraction unbound in plasma; fa, fraction absorbed; k_a,_ absorption rate constant; fu_gut,_ fraction unbound in gut enterocytes; Q_gut_, hybrid parameter representing drug absorption rate from the gut lumen, removal of drug from the enterocyte by enterocytic blood supply, and enterocyte volume; V_SS_, steady-state volume of distribution; Kp Scalar, tissue to plasma ratio; CL_R_, renal clearance; CL_PDM_, maternal-placental barrier; CL_PDF_, placenta-fetal barrier; PSA, polar surface area; HBD, hydrogen bond donor.

**Table 2:** Predicted pharmacokinetic parameters of escitalopram in nonpregnant and pregnant women at different gestational weeks following multiple oral doses.

Dose (mg)	Gestational Week	Predicted Escitalopram Parameters		
		C_max_ (ng/mL)	T_max_ (h)	AUC (ng·h/mL)
10	0	18.38 (-)	4.00	364.05 (-)
	20	13.70 (↓25.46%)	3.96	268.98 (↓26.11%)
	35	10.46 (↓43.09%)	3.27	204.14 (↓43.92%)
20	0	36.76 (-)	4.01	728.11 (-)
	20	27.39 (↓25.49%)	3.97	537.97 (↓26.11%)
	35	20.93 (↓43.06%)	3.27	408.28 (↓43.93%)

All predicted parameters are presented as the arithmetic mean. Percentages in parentheses indicate change relative to gestational week 0. Downward arrows denote a decrease. T_max_ values are presented as arithmetic means only. Abbreviations: C_max_, maximum plasma concentration; T_max_, time to maximum plasma concentration; AUC, area under the plasma concentration–time curve.

**Table 3:** Predicted pharmacokinetic parameters of escitalopram in pregnant women based on CYP2C19 phenotypes after multiple 10 mg oral doses.

CYP2C19 Phenotype	Gestational Week	Predicted Escitalopram Parameters		
		C_max_ (ng/mL)	T_max_ (h)	AUC (ng·h/mL)
IM	35	11.57	3.31	227.88
NM	40	10.52	3.06	202.49

Abbreviations: C_max_, maximum plasma concentration; T_max_, time to maximum plasma concentration; AUC, area under the plasma concentration–time curve; IM, intermediate metabolizer; NM, normal metabolizer.

## Data Availability

The data presented in this study are available in this article/supplementary information.

## Supplementary Materials

The following supporting information can be downloaded at https://www.mdpi.com/article/10.3390/pharmaceutics17101341/s1, Figure S1: Predicted and observed plasma concentration–time profiles of escitalopram in nonpregnant women after (a,b) a single 20-mg oral dose, (c) multiple 10-mg oral doses, and (d) multiple 30-mg oral doses; Figure S2: Absolute mean (µ*) values of (a–d) C_max_ and (e–h) AUC of escitalopram in the nonpregnant model after (a,b,e,f) a single oral dose of 20 mg, (c,g) multiple oral doses of 10 mg, and (d,h) multiple oral doses of 30 mg; Figure S3: Predicted umbilical vein concentration-time profiles of escitalopram at term in pregnant women following multiple oral doses of (a) 10 mg, (b) 20 mg; Figure S4: Predicted umbilical vein concentration–time profiles of escitalopram at term in pregnant women following multiple oral doses of 10 mg based on the CYP2C19 normal metabolizer phenotype; Figure S5: Predicted plasma concentration–time profiles of escitalopram in a pregnant women with CYP2C19 PM, CYP3A4 PM, and CYP2D6 IM phenotypes following multiple oral doses of 20 mg at (a) gestational week 0, (b) week 20, and (c) week 35; Figure S6: Predicted escitalopram plasma concentration–time profiles at gestational week 35 for CYP2C19 and CYP2D6 phenotype combinations (20 mg/day); Table S1: Predicted and observed pharmacokinetic parameters, along with prediction ratios following single or multiple oral administration of escitalopram in nonpregnant women; Table S2: Predicted pharmacokinetic parameters of escitalopram in term pregnant women after multiple oral doses; Table S3: Predicted escitalopram pharmacokinetic parameters of escitalopram in term pregnant women after multiple 10-mg oral doses, based on the CYP2C19 normal metabolizer phenotype; Table S4: Summary of escitalopram dose optimization during pregnancy based on CYP2C19 phenotypes, Table S5: Predicted escitalopram exposure at gestational week 35 for CYP2C19 and CYP2D6 phenotype combinations (20 mg/day).

## Abbreviations

The following abbreviations are used in this manuscript:

PBPK	Physiologically based pharmacokinetic
C_max_	Maximum plasma concentration
AUC	Area under the curve
B/P	Blood to plasma ratio
f_a_	Fraction absorbed
f_u,gut_	Fraction unbound in gut enterocytes
CL_R_	Renal clearance
k_a_	Absorption rate constant
Q_gut_	Hybrid parameter representing drug absorption rate from the gut lumen, removal of drug from the enterocyte by enterocytic blood supply, and enterocyte volume
K_p_ scalar	Tissue to plasma partition coefficient
V_ss_	Steady state volume of distribution
GW	Gestational week
CL_PDM_	Maternal-placental barrier
CL_PDF_	Placental-fetal barrier
IM	Intermediate metabolizer
NM	Normal metabolizer
UM	Ultrarapid metabolizer
PM	Poor metabolizer
