# Supplementary material for: Escitalopram Dose Optimization During Pregnancy: A PBPK Modeling Approach
Source: Pharmaceutics. 2025 Oct 17;17(10):1341. doi: 10.3390/pharmaceutics17101341 (PMC12567279; doi:10.3390/pharmaceutics17101341)
Supplement: Supplementary file 1 [file pharmaceutics-17-01341-s001.zip › pharmaceutics-3894231-supplementary.pdf]

Supplementary materials

Application of Physiologically Based Pharmacokinetic Modeling to Inform Escitalopram Dosing Recommendations During Pregnancy

Seo-Yeon Choi[1], Eunsol Yang[2], Kwang-Hee Shin, PhD[1,3*]

[1]College of Pharmacy, Research Institute of Pharmaceutical Sciences, Kyungpook National University, Daegu 41566, Republic of Korea

[2] Department of Bioengineering and Therapeutic Sciences, University of California, San Francisco, San Francisco, CA, United States of America

[3]Infectious Disease Healthcare, Kyungpook National University, Daegu, 41566, Republic of Korea.

List of Supplementary Tables

Table S1 Predicted and observed pharmacokinetic parameters along with prediction ratios after single or multiple oral administration of escitalopram in nonpregnant women

Table S2 Predicted pharmacokinetic parameters in pregnant women at term after multiple oral doses of escitalopram

Table S3 Predicted escitalopram pharmacokinetic parameters in pregnant women at term following multiple oral doses of 10 mg based on the CYP2C19 normal metabolizer phenotype

Table S4 Summary of escitalopram dose optimization during pregnancy based on

CYP2C19 phenotypes.

Table S1. Predicted and observed pharmacokinetic parameters along with prediction ratios after single or multiple oral administration of escitalopram in nonpregnant women.

References	Dose	C_{max}		Prediction ratio	T_{max} (h)		AUC (ng·h/mL)		Prediction ratio
		Observed	Predicted		Observed	Predicted	Observed	Predicted	
[23]	20 mg	58.0 nM (44.1-74.4)	48.16 nM (30.7-68.9)	0.83	3.0 (1.5-4.5)	5.70 (3.1-9.7)	1772 (789-2755)	2026.35 (924.6-3443.1)	1.14
[22]	20 mg	18.8 ng/mL (14.4-23.3)	15.65 ng/mL (10.01-21.99)	0.83	3.0 (1.5-4.5)	5.55 (3.2-8.8)	637 (281-993)	651.82 (314.6-1093.8)	1.02
	10 mg	20.6 ng/mL (10.3-30.9)	16.80 ng/mL (8.90-26.16)	0.82	3.9 (2.1-5.7)	3.98 (2.9-4.9)	360.2 (141.5-578.9)	330.14 (157.8-551.7)	0.92
	30 mg	64.4 ng/mL (30.7-98.1)	50.87 ng/mL (26.65-78.66)	0.79	4.1 (1.4-6.8)	3.98 (2.8-5.0)	1100.9 (367.3-1834.5)	1000.20 (471.3-1672.7)	0.91

All parameters are presented as the arithmetic mean (5th-95th percentile) except prediction ratio, which is presented as the arithmetic mean (95% confidential interval). C_{max}, maximum plasma concentration; T_{max}, time to maximum plasma concentration; AUC, area under the plasma concentration–time curve.

Table S2. Predicted pharmacokinetic parameters in pregnant women at term after multiple oral doses of escitalopram.

Dose	Predicted umbilical vein PK parameters			
	C_{max} (ng/mL)	T_{max} (h)	AUC (ng·h/mL)	Cord/Maternal ratio
10 mg	7.27	4.28	135.92	0.71
20 mg	14.40	4.29	269.15	0.69

C_{max}, maximum plasma concentration; T_{max}, time to maximum plasma concentration; AUC, area under the plasma concentration-time curve.

Table S3. Predicted escitalopram pharmacokinetic parameters in pregnant women at term following multiple oral doses of 10 mg based on the CYP2C19 normal metabolizer phenotype.

Dose	Predicted umbilical vein PK parameters			
	C_{max} (ng/mL)	T_{max} (h)	AUC (ng·h/mL)	Cord/Maternal ratio
10 mg	7.52	4.35	140.60	0.70

C_{max}, maximum plasma concentration; T_{max}, time to maximum plasma concentration; AUC, area under the plasma concentration-time curve.

Table S4. Summary of escitalopram dose optimization during pregnancy based on CYP2C19 phenotypes.

**CYP2C19 phenotype**		**UM**			**NM**			**IM**			**PM**		
**Trimester**		**First**	**Second**	**Third**	**First**	**Second**	**Third**	**First**	**Second**	**Third**	**First**	**Second**	**Third**
$C_{min,ss}$ (ng/mL)	10 mg	7.25	6.02	5.23	9.07	7.36	6.23	9.07	7.26	6.07	12.0	9.42	7.67
	20 mg	14.5	12.0	10.5	18.1	14.7	12.5	18.1	14.5	12.1	24.1	18.8	15.3
$C_{max,ss}$ (ng/mL)	10 mg	13.1	10.7	9.04	15.5	12.5	10.4	15.8	12.6	10.4	19.2	15.1	12.2
	20 mg	26.2	21.5	18.0	31.0	25.0	20.7	31.5	25.2	20.7	38.3	30.2	24.4
$C_{avg,ss}$ (ng/mL)	10 mg	10.3	8.6	7.2	12.5	10.2	8.4	12.6	10.1	8.3	15.9	12.5	10.1
	20 mg	20.7	17.1	14.5	25.0	20.3	16.9	25.2	20.2	16.5	31.8	25.1	20.2

UM, ultrarapid metabolizer; NM, normal metabolizer; IM, intermediate metabolizer; PM, poor metabolizer; $C_{min,ss}$, minimum plasma concentration at steady state; $C_{max,ss}$, maximum plasma concentration at steady state; $C_{avg,ss}$, average plasma concentration at steady state.

List of Supplementary Figure

Figure S1 Predicted and observed plasma concentration-time profiles of escitalopram after (a, b) a single oral dose of 20 mg, (c) multiple oral doses of 10 mg, and (d) multiple oral doses of 30 mg

Figure S2 Predicted umbilical vein concentration–time profiles of escitalopram at term in pregnant women following multiple oral doses of (a) 10 mg, (b) 20 mg

Figure S3 Predicted umbilical vein concentration-time profiles of escitalopram at term in pregnant women following multiple oral doses of 10 mg based on the CYP2C19 normal metabolizer phenotype

Figure S4 Predicted plasma concentration–time profiles of escitalopram in a pregnant women with CYP2C19 PM, CYP3A4 PM, and CYP2D6 IM phenotypes following multiple oral doses of 20 mg at (a) gestational week 0, (b) week 20, and (c) week 35

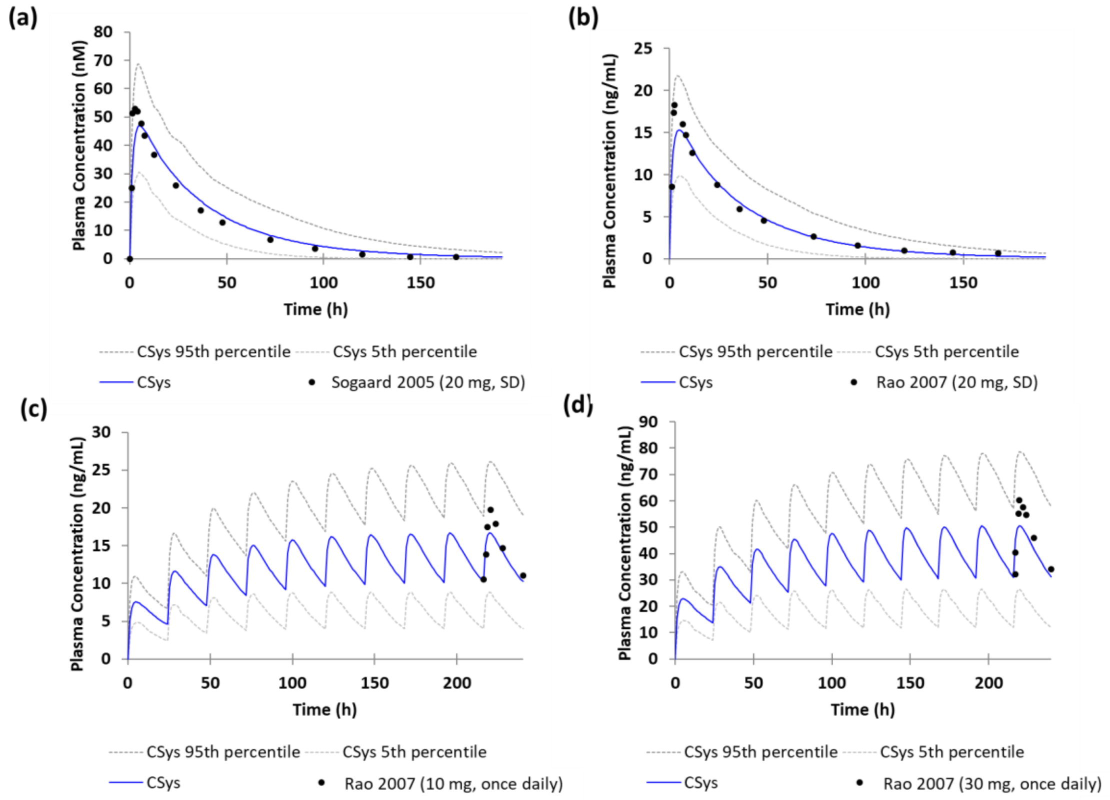

Figure S1 Predicted and observed plasma concentration-time profiles of escitalopram after (a, b) a single oral dose of 20 mg, (c) multiple oral doses of 10 mg, and (d) multiple oral doses of 30 mg. Blue line indicates the mean of predicted plasma concentrations and gray dotted lines indicate the 5th and 95th percentiles of predicted plasma concentrations. Circles indicate the mean of observed plasma concentrations, respectively. SD: single dose.

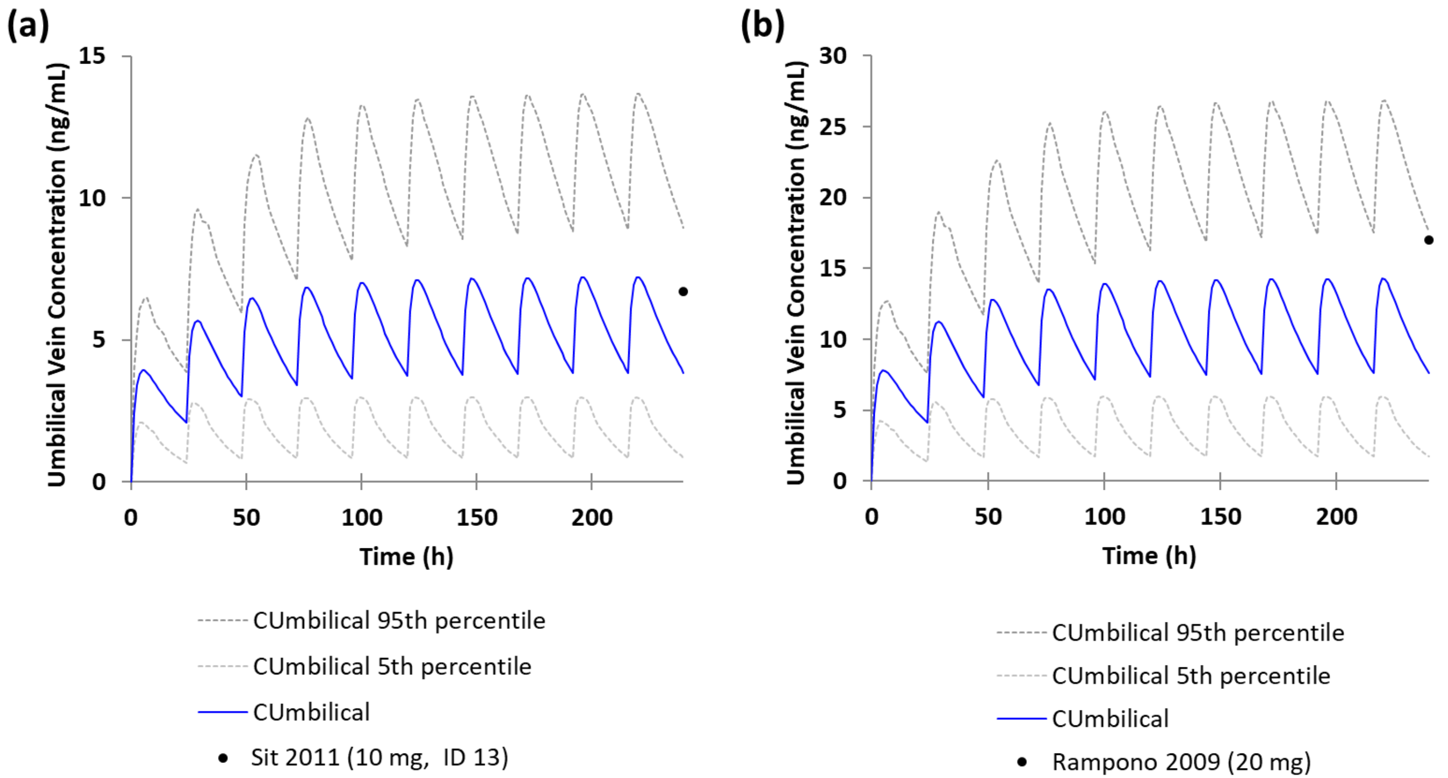

Figure S2. Predicted umbilical vein concentration–time profiles of escitalopram at term in pregnant women following multiple oral doses of (a) 10 mg, (b) 20 mg. Blue line indicates the mean of predicted umbilical vein concentrations and gray dotted lines indicate the 5th and 95th percentiles of predicted umbilical vein concentrations. Circle indicates observed umbilical vein concentration, respectively.

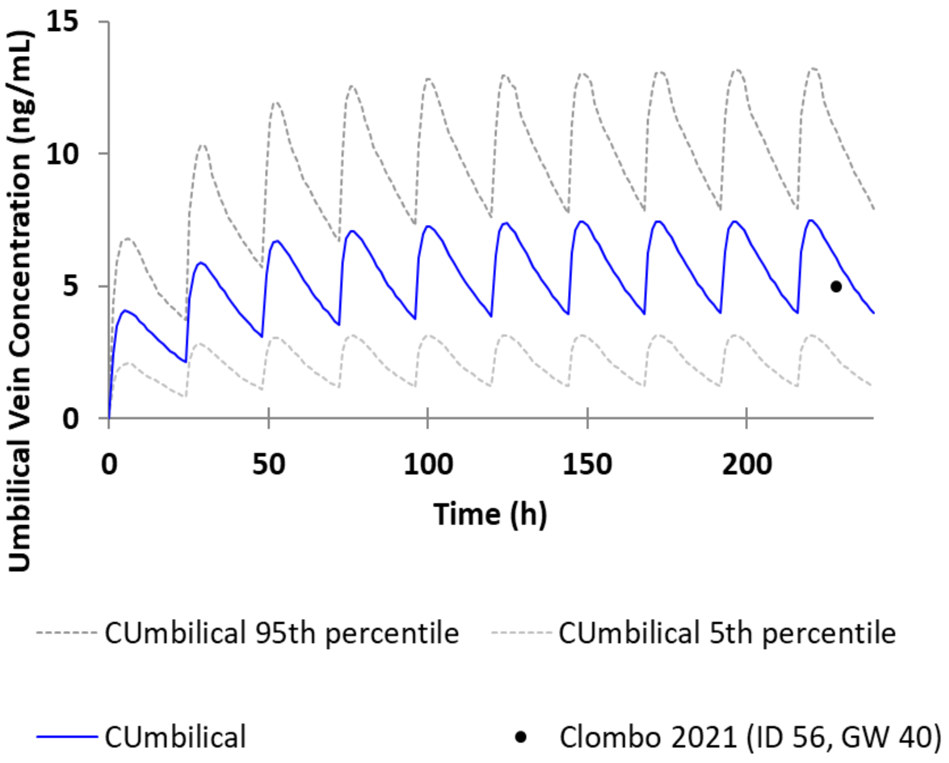

Figure S3. Predicted umbilical vein concentration-time profiles of escitalopram at term in pregnant women following multiple oral doses of 10 mg based on the CYP2C19 normal metabolizer phenotype. Blue line indicates the mean of predicted umbilical vein concentrations and gray dotted lines indicate the 5th and 95th percentiles of predicted umbilical vein concentrations. Circle indicates observed umbilical vein concentration.

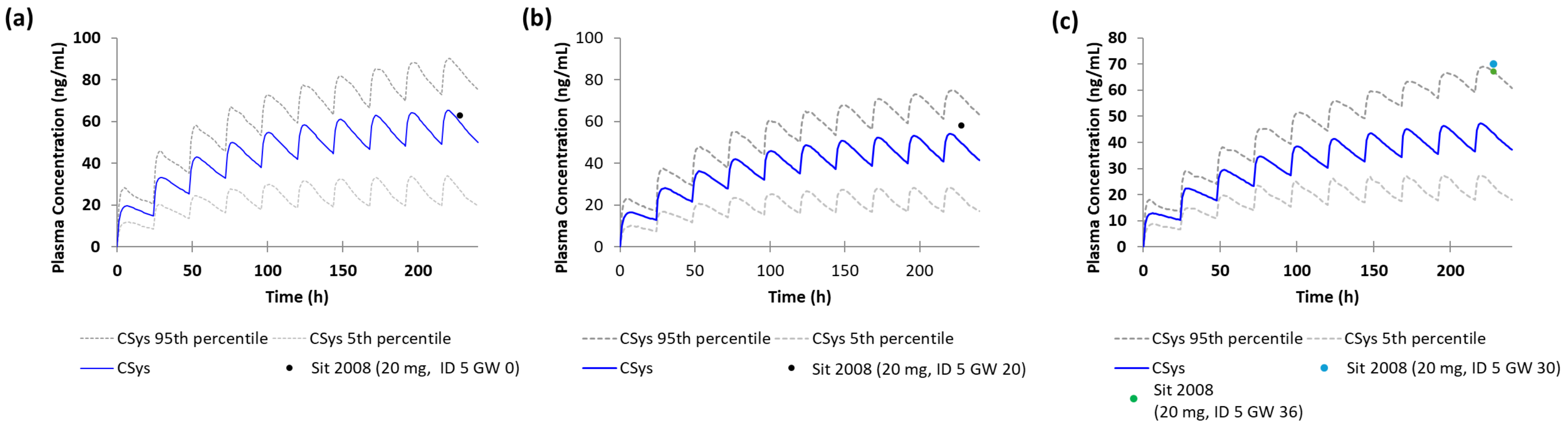

Figure S4. Predicted plasma concentration–time profiles of escitalopram in a pregnant women with CYP2C19 PM, CYP3A4 PM, and CYP2D6 IM phenotypes following multiple oral doses of 20 mg at (a) gestational week 0, (b) week 20, and (c) week 35. Blue line indicates the mean of predicted plasma concentrations and gray dotted lines indicate the 5th and 95th percentiles of predicted plasma concentrations. Circles indicate observed plasma concentrations, respectively. PM: poor metabolizer; IM: intermediate metabolizer.
